# Supplementary material for: Clinical Utility of Next-Generation Sequencing-Based Panel Testing under the Universal Health-Care System in Japan: A Retrospective Analysis at a Single University Hospital
Source: Cancers (Basel). 2021 Mar 5;13(5):1121. doi: 10.3390/cancers13051121 (PMC7961835; doi:10.3390/cancers13051121)
Supplement: Supplementary file 1 [file cancers-13-01121-s001.pdf]

cancers

MDPI

Supplementary Materials

Clinical utility of Next-Generation Sequencing-Based Panel Testing under the Universal Health Care System in Japan: A Retrospective Analysis at a Single University Hospital

Chiaki Inagaki, Daichi Maeda, Kazue Hatake, Yuki Sato, Kae Hashimoto, Daisuke Sakai, Shinichi Yachida, Iwao Nonomura and Taroh Satoh

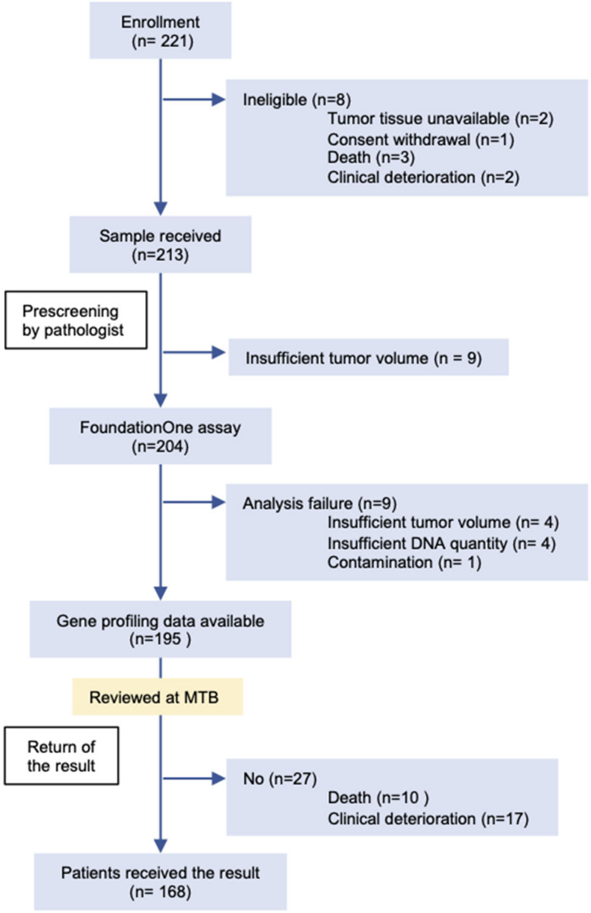

Figure S1. CONSORT diagram of patients enrolled in the study. MTB; molecular tumor board.

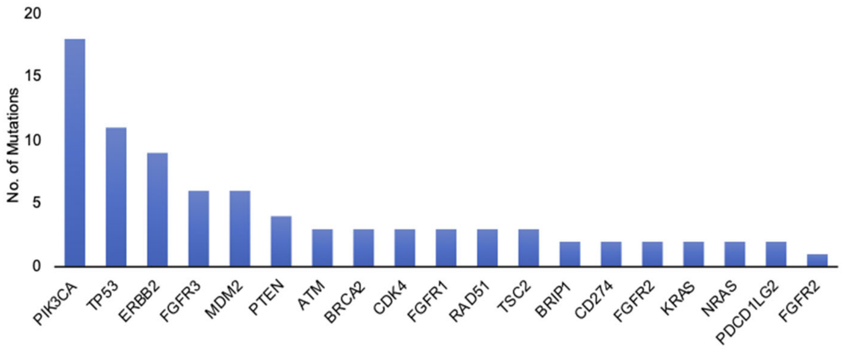

Figure S2. Top 20 frequent genomic alterations with the treatment recommendation.

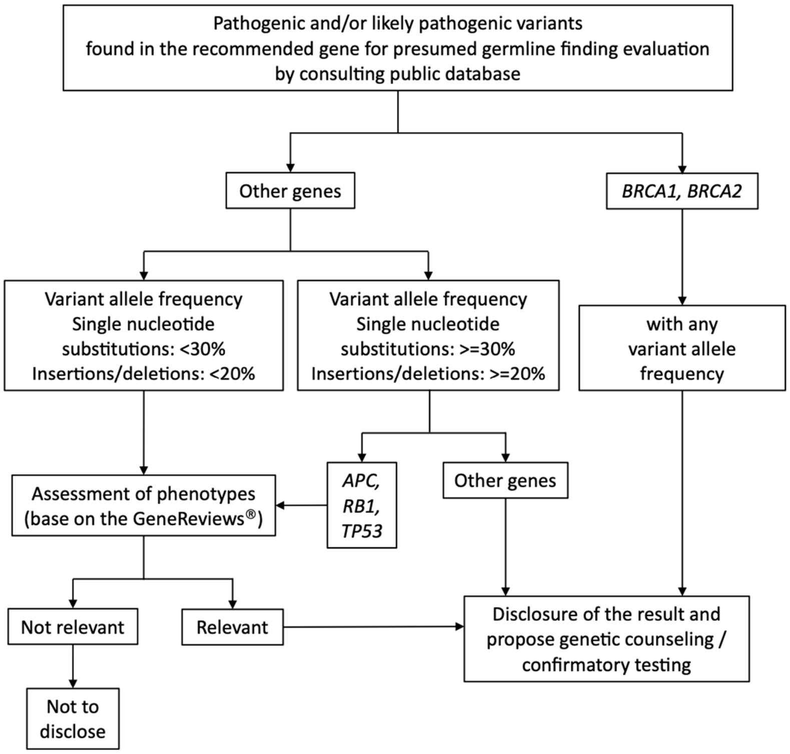

Figure S3. The operation workflow for evaluating and nominating presumed germline findings from tumor-only sequencing panel

The workflow is adapted from the proposal of the Japan Agency for Medical Research and Development (AMED) study group concerning the information transmission process in genomic medicine). *APC*; adenomatous polyposis coli, *BRCA1*; breast cancer susceptibility gene 1, *BRCA2*; breast cancer susceptibility gene 2, *RB1*; retinoblastoma 1, *TP53*; tumor protein P53

Table S1. Actionable alterations according to cancer type.

Cancer Type	Patient, n	No. of patients with actionable mutation, n (%)	No. of actionable mutation, n	Level of Evidence, n (%)					
				1	2	3A	3B	4	Other
Total	168	70 (64.8)	107	9 (8.4)	6 (5.6)	6 (5.6)	44 (41.1)	17 (15.9)	25 (23.4)
Colorectal	45	19 (42.4)	26	1 (3.8)	3 (11.5)	0 (0)	8 (30.8)	3 (11.5)	11 (42.3)
Sarcoma	22	8 (36.4)	16	1 (6.3)	1 (6.3)	1 (6.3)	11 (68.8)	1 (6.3)	1 (6.3)
Pancreatic	18	4 (22.2)	7	0 (0)	0 (0)	1 (14.3)	2 (28.6)	1 (14.3)	3 (42.9)
Gastric	13	2 (15.4)	4	0 (0)	0 (0)	0 (0)	1 (25.0)	2 (50.0)	1 (25.0)
Ovarian	11	5 (35.5)	6	1 (16.7)	0 (0)	0 (0)	3 (50.0)	0 (0)	2 (33.3)
Bile Duct	9	5 (55.6)	6	0 (0)	0 (0)	0 (0)	5 (83.3)	1 (16.7)	0 (0)
Esophageal	8	4 (50.0)	6	0 (0)	0 (0)	0 (0)	3 (50.0)	0 (0)	3 (50.0)
Breast	7	6 (85.7)	10	6 (60.0)	0 (0)	1 (10.0)	0 (0)	3 (30.0)	0 (0)
Cervical	6	2 (33.3)	3	0 (0)	0 (0)	0 (0)	1 (33.3)	0 (0)	2 (66.7)
Small intestinal	5	4 (80.0)	4	0 (0)	0 (0)	0 (0)	4 (100.0)	0 (0)	0 (0)
Endometrial	3	3 (100.0)	5	0 (0)	0 (0)	0 (0)	3 (60.0)	1 (20.0)	1 (20.0)
Non-Small Cell Lung	3	2 (66.6)	5	0 (0)	1 (20.0)	1 (20.0)	0 (0)	2 (40.0)	1 (20.0)
Brain	3	2 (66.6)	4	0 (0)	1 (25.0)	0 (0)	2 (50.0)	1 (25.0)	0 (0)
Melanoma	3	2 (66.6)	2	0 (0)	0 (0)	2 (100.0)	0 (0)	0 (0)	0 (0)
Unknown Primary	3	1 (33.3)	1	0 (0)	0 (0)	0 (0)	0 (0)	1 (100.0)	0 (0)
Hepatocellular Carcinoma	3	1 (33.3)	2	0 (0)	0 (0)	0 (0)	1 (50.0)	1 (50.0)	0 (0)
Neuroblastoma	3	0 (0)	-	-	-	-	-	-	-
Kidney	1	0 (0)	-	-	-	-	-	-	-
Prostate	1	0 (0)	-	-	-	-	-	-	-
Urinary Tract	1	0 (0)	-	-	-	-	-	-	-

Table S2. List of cases that underwent genomically matched treatment beyond standard of care based on MTB recommendation.

Cancer Type	Age	Sex	Targeted gene	LE	Treatment	Institution	Clinical Benefit
Endometrial	62	F	*ATM*	3B	Clinical trial; ATR inhibitor	Outside hospital	NA
			JAK1	Other	Clinical trial; JAK inhibitor	Our hospital	NA
Colorectal	43	F	TMB-H	1	Clinical trial; PD-1 inhibitor	Our hospital	NA
Gastric	68	M	*FGFR2* amp	4	Clinical trial; FGFR2 inhibitor	Our hospital	NA
Bile duct	69	M	*FGFR2* amp	4	Clinical trial; FGFR2 inhibitor	Our hospital	NA
Bile duct	75	F	*ERBB2* amp	3B	Off label; Trastuzumab/Pertuzumab	Our hospital	Yes
					Off label; Trastuzumab deruxtecan	Our hospital	Yes
Colorectal	59	F	*ERBB2* amp	2	Off label; Trastuzumab/Pertuzumab	Our hospital	No

amp; amplification, *ATM*; ataxia telangiectasia mutated, *ERBB2*; erbB-2 receptor tyrosine-protein kinase, *FGFR2*; fibroblast growth factor receptor 2, *JAK1*; janus kinase 1, MTB; molecular tumor board, TMB-H; tumor mutation burden high, LE; level of evidence.

Table S3. Patients' preference for receiving presumed germline finding.

Wanting to receive presumed germline finding*	Yes (n)	166
	No (n)	2
Sharing information with family members**	Yes (n)	156
	No (n)	8

*For minor patients, answers are obtained from their parents/guardians with patients' assent. **exclude minor patients.

Table S4. Presumed germline finding gene list used for assessing F1CDx.

Gene	Major Phenotype
APC	FAP
ATM	Breast cancer
BAP1	Malignant Mesothelioma etc.
BRCA1	HBOC
BRCA2	HBOC
BRIP1	Ovarian cancer
CDH1	Diffuse gastric cancer
CDK4	Melanoma
CDKN2A	Melanoma/Pancreatic cancer
CHEK2	Breast cancer
FH	Hereditary Leiomyomatosis and Renal Cell cancer
FLCN	Birt-Hogg-Dube syndrome
MEN1	MEN1
MET	GIST
MLH1	Lynch syndrome
MSH2	Lynch syndrome
MSH6	Lynch syndrome
MUTYH	MAP

NBN	Breast cancer
NF1	NF1
NF2	NF2
PALB2	Breast cancer
PMS2	Lynch syndrome
POLD1	Colon cancer
POLE	Colon cancer
PTEN	*PTEN* hamartoma
RAD51C	Ovarian cancer
RAD51D	Ovarian cancer
RB1	Retinoblastoma
RET	MEN2
SDHA	HPPS
SDHAF2	HPPS
SDHB	HPPS
SDHC	HPPS
SDHD	HPPS
SMAD4	Juvenile Polyposis
STK11	Peutz-Jeghers syndrome
TGFBR2	Loeys-Dietz syndrome
TP53	Li-Fraumeni syndrome
TSC1	Tuberous Sclerosis
TSC2	Tuberous Sclerosis
VHL	VHL
WT1	*WT1*-related Wilms tumor

APC; adenomatous polyposis coli, *ATM*; ataxia telangiectasia mutated, *BAP1*; *BRCA1* associated protein 1, *BRCA1*; breast cancer susceptibility gene1, *BRCA2*; breast cancer susceptibility gene2, *BRIP1*; *BRCA1* interacting protein C-terminal helicase 1, *CDH1*; cadherin-1, *CDK4*; cyclin dependent kinase 1, *CDKN2A*; cyclin Dependent Kinase Inhibitor 2A, *CHEK2*; checkpoint kinase 2, FAP; familial adenomatous polyposis, *FH*; fumarate hydratase, *FLCN*; folliculin, GIST; gastrointestinal stromal tumor, HBOC; hereditary breast and ovarian cancer, HPPS; hereditary pheochromocytoma/ paraganglioma syndrome, MAP;*MUTYH*-Associated polyposis, *MEN1*; multiple endocrine neoplasia type1, *MEN2*; multiple endocrine neoplasia type2, *MET*; hepatocyte growth factor receptor, *MLH1*; *MutL* homolog 1, *MSH2*; *MutS* Homolog 2, *MSH6*; *mutS* homolog 6, *MUTYH*; *mutY* homolog, *NBN*; nibrin, *NF1*; neurofibromatosis type1, *NF2*; neurofibromatosis type2, *PALB2*; partner and localizer of *BRCA2*, *PMS2*; *PMS1* homolog 2, *POLD1*; DNA polymerase delta 1, *POLE*; DNA polymerase epsilon 1, *PTEN*; phosphatase and tensin homolog, *RAD51C*; *RAD51* homolog C, *RAD51D*; *RAD51* homolog D, *RB1*; retinoblastoma 1, *RET*; rearranged during transfection, *SDHA*; succinate dehydrogenase complex flavoprotein subunit A, *SDHAF2*; succinate dehydrogenase complex assembly factor 2, *SDHB*; succinate dehydrogenase complex subunit B, *SDHC*; succinate dehydrogenase complex subunit C, *SDHD*; succinate dehydrogenase complex subunit D, *SMAD4*; mothers against decapentaplegic homolog 4, *STK11*; serine/threonine kinase 11, *TGFBR2*; transforming growth factor beta receptor 2, *TP53*; tumor protein P53, *TSC1*; tuberous sclerosis complex 1,*TSC2*; tuberous sclerosis complex 2, *VHL*; von Hippel-Lindau, *WT1*; Wilms' tumor 1,.
